# Dignity of Risk Education With Healthcare Staff: Preliminary Results, Lessons Learned, and Future Directions

**DOI:** 10.1093/geroni/igaf122.2090

**Published:** 2025-12-31

**Authors:** Catherine-Anne Murray, Elizabeth Gillis, Connor Dawes, Caroline King, Karen Nicholls, Kailey Durette

**Affiliations:** Nova Scotia Health, Halifax, Nova Scotia, Canada; Nova Scotia Health, Halifax, Nova Scotia, Canada; Nova Scotia Health Implementation Science, Halifax, Nova Scotia, Canada; Nova Scotia Health Implementation Science, Halifax, Nova Scotia, Canada; Nova Scotia Health, Halifax, Nova Scotia, Canada; Nova Scotia Health, Nova Scotia, Canada

## Abstract

Following the Dignity of Risk (DoR) adult participatory education workshops with healthcare staff, post workshop surveys were completed by over 200 participants (response rate ∼30%). The aims were: 1. To measure effectiveness of in person education with healthcare staff regarding the application of a DoR approach in the care of older adults living with frailty. 2. Use key findings and lessons learned to further inform education methods and implications to healthcare policy and quality improvement. Results showed an increase in staff confidence in applying DoR into practice and having DoR and risk management conversations with caregivers. Several main themes emerged as “takeaways”: staff perceived older adults often value autonomy over safety, risk can be included as a part of a supportive care plan, and the importance of a balanced, broadened and strength-based approach to risk management in care planning. Additionally, staff identified further learning needs to enhance skills in risk management care planning and conversations. There were notable lessons learned that continue to guide DoR initiatives, including an inpatientpilot with DoR/Frailty experts embedded onsite and supporting staff. Knowledge translation and staff behaviour change were measured via the Behaviour Change Wheel methods, and the Consolidated Framework for Implementation Research. In terms of behaviour change in practice that leads to measurable outcomes for older adults, engaged leadership (managers and team leads) is required to support and reinforce this culture shift. Additionally, enhanced interprofessional collaboration that focuses on a united approach to prioritize autonomy of the older adult first, followed closely by risk management. Policies (i.e. Falls Prevention) and guidelines need to incorporate human rights language and strength-based risk management considerations. Only when healthcare staff feel competent and empowered to support positive risk with older adults, will those older adults likely be able to return home from hospital or remain home longer despite living with frailty and changing abilities.

